# Utility of CSF Cytokine/Chemokines as Markers of Active Intrathecal Inflammation: Comparison of Demyelinating, Anti-NMDAR and Enteroviral Encephalitis

**DOI:** 10.1371/journal.pone.0161656

**Published:** 2016-08-30

**Authors:** Kavitha Kothur, Louise Wienholt, Shekeeb S. Mohammad, Esther M. Tantsis, Sekhar Pillai, Philip N. Britton, Cheryl A. Jones, Rajeshwar R. Angiti, Elizabeth H. Barnes, Timothy Schlub, Sushil Bandodkar, Fabienne Brilot, Russell C. Dale

**Affiliations:** 1 Brain Autoimmunity group, Institute for Neuroscience and Muscle Research, The Children’s Hospital at Westmead, University of Sydney, Sydney, NSW, Australia; 2 Department of Clinical Immunology, Royal Prince Alfred Hospital, Sydney, NSW, Australia; 3 Marie Bashir Institute for Infectious Diseases and Biosecurity, University of Sydney, and The Children’s Hospital at Westmead, Sydney, NSW, Australia; 4 Department of Neonatology, Nepean Hospital, University of Sydney, Sydney, NSW, Australia; 5 NHMRC Clinical Trials Centre, University of Sydney, Sydney, NSW, Australia; 6 Sydney School of Public Health, Sydney Medical School, University of Sydney, Sydney, NSW, Australia; 7 Department of Biochemistry, The Children’s Hospital at Westmead, Sydney, NSW, Australia; Ludwig-Maximilians-Universitat Munchen, GERMANY

## Abstract

**Background:**

Despite the discovery of CSF and serum diagnostic autoantibodies in autoimmune encephalitis, there are still very limited CSF biomarkers for diagnostic and monitoring purposes in children with inflammatory or autoimmune brain disease. The cause of encephalitis is unknown in up to a third of encephalitis cohorts, and it is important to differentiate infective from autoimmune encephalitis given the therapeutic implications.

**Aim:**

To study CSF cytokines and chemokines as diagnostic biomarkers of active neuroinflammation, and assess their role in differentiating demyelinating, autoimmune, and viral encephalitis.

**Methods:**

We measured and compared 32 cytokine/chemokines using multiplex immunoassay and APRIL and BAFF using ELISA in CSF collected prior to commencing treatment from paediatric patients with confirmed acute disseminated encephalomyelitis (ADEM, n = 16), anti-NMDAR encephalitis (anti-NMDAR E, n = 11), and enteroviral encephalitis (EVE, n = 16). We generated normative data using CSF from 20 non-inflammatory neurological controls. The sensitivity of CSF cytokine/chemokines to diagnose encephalitis cases was calculated using 95^th^ centile of control values as cut off. We correlated CSF cytokine/chemokines with disease severity and follow up outcome based on modified Rankin scale. One-way hierarchical correlational cluster analysis of molecules was performed in different encephalitis and outcome groups.

**Results:**

In descending order, CSF TNF-α, IL-10, IFN-α, IL-6, CXCL13 and CXCL10 had the best sensitivity (>79.1%) when all encephalitis patients were included. The combination of IL-6 and IFN-α was most predictive of inflammation on multiple logistic regression with area under the ROC curve 0.99 (CI 0.97–1.00). There were no differences in CSF cytokine concentrations between EVE and anti-NMDAR E, whereas ADEM showed more pronounced elevation of Th17 related (IL-17, IL-21) and Th2 (IL-4, CCL17) related cytokine/chemokines. Unlike EVE, heat map analysis showed similar clustering of cytokine/chemokine molecules in immune mediated encephalitis (ADEM and anti-NMDAR E). Th1 and B cell (CXCL13 and CXCL10) molecules clustered together in patients with severe encephalopathy at admission and worse disability at follow up in all encephalitis. There was no correlation between CSF neopterin and IFN-γ or IFN-α.

**Conclusion:**

A combination panel of cytokine/chemokines consisting of CSF TNF-α, IL-10, IFN-α, IL-6, CXCL13 and CXCL10 measured using multiplex immunoassay may be used to diagnose and monitor intrathecal inflammation in the brain. Given their association with worse outcome, certain key chemokines (CXCL13, CXCL10) could represent potential therapeutic targets in encephalitis.

## Introduction

Paediatric neuroinflammatory disorders represent a heterogeneous group of disorders including encephalitis, demyelinating disorders and other immune-mediated autoinflammatory CNS disorders [1]. It is important to differentiate neuroinflammatory disorders from other neurological conditions, as the former are potentially treatable. There is a need to develop CSF biomarkers in neuroinflammatory disorders of the brain to determine if persistent neurological symptoms are due to active ongoing CNS inflammation or alternatively to residual brain injury [2, 3]. Currently available investigations do not have high sensitivity in detecting neuroinflammation, for example MRI is frequently normal in autoimmune encephalitis. The currently available CSF markers including pleocytosis, neopterin, oligoclonal bands lack sensitivity and specificity, and cell neuronal surface antibodies are found only in a small proportion of neuroinflammatory conditions [4–8]. The etiology of 37–63% patients with encephalitis remains elusive in encephalitis cohort studies despite extensive investigations, and it can be difficult to differentiate infective from autoimmune encephalitis [4, 9–11].

Cytokines and chemokines are key intercellular mediators in inflammation and have been shown to be elevated in a number of inflammatory disorders of the brain (S1 File: Table C). Naïve T cells differentiate into Th1, Th2, Th17, Treg, and Tfh cells depending on the nature of antigenic stimulation and surrounding cytokine milieu. Even though cytokine/chemokines exhibit multiple effects on a range of inflammatory cells and have overlapping functions, most of them exhibit unique properties and are elevated in many neuroinflammatory disorders of brain, which support their role as potential biomarkers [1, 12]. Previous studies of CSF cytokine/chemokines in autoimmune encephalitis and ADEM are limited [1].

In order to improve our CSF diagnostic repertoire, we studied the use of CSF cytokines and chemokines in well-defined encephalitis syndromes, and examined the ability of these molecules to differentiate demyelinating, autoimmune and viral encephalitis etiologies.

## Patients and Methods

As part of the ethically approved protocol, we wrote to all parents and gained written consent to use the stored CSF for this study. This study was approved by HREC of Sydney Children’s Hospital network (LNRSSA/14/SCHN/283).

### Patient Characteristics

We included patients with encephalitis who had stored frozen acute CSF available, using neurology and neuroimmunology clinical databases at the Children's Hospital at Westmead, Sydney, Australia between 2006 and 2014 [9]. The cases of inflammatory demyelinating encephalitis (acute disseminated encephalomyelitis, ADEM), autoimmune encephalitis (anti-NMDAR encephalitis, anti-NMDAR-E) and viral encephalitis (enteroviral encephalitis, EVE) were rigorously selected based on clinical and lab diagnostic criteria. All patients with encephalitis fulfilled the consensus criteria for definition of encephalitis which is defined as encephalopathy plus two or more of the following: fever (temperature ≥38°C), seizure(s), focal neurological findings, pleocytosis (>5 WBCs/mL), and electroencephalogram (EEG) or/and neuroimaging findings compatible with encephalitis [13]. Patients with ADEM (n = 16) fulfilled International Pediatric MS Study Group (IPMSSG) diagnostic criteria 2013 [14]. Fifteen of the ADEM patients underwent serum MOG (Myelin oligodendrocyte glycoprotein) antibody analysis, 10 of the ADEM patients were MOG antibody positive and 5 were MOG antibody negative [15]. The diagnosis of anti-NMDAR encephalitis (n = 11) was based on characteristic clinical features of psychiatric and cognitive features, movement disorder and sleep disturbances along with the presence of NMDAR antibodies in CSF (n = 10) / serum (n = 9). In patients with EVE (n = 16), enterovirus infection was confirmed by nucleic acid detection in the CSF (confirmed EV encephalitis, n = 6) and other sites including throat or stool or nasopharyngeal aspirate using polymerase chain reaction (possible EV encephalitis, n = 9), or by serological evidence (possible, n = 1) [16].The clinical features, investigations, time of CSF sample collection from onset of neurological symptoms (CSF timing), treatment and outcome are presented in Table 1. The nadir neurological disability during the acute phase at admission and follow up were calculated from the patient notes retrospectively using modified Rankin scale (MRS), as previously [17].

**Table 1:** Clinical, investigations, treatment and outcome details of ADEM, anti-NMDAR encephalitis and enteroviral encephalitis cohorts.

Clinical syndrome (n)	ADEM (16)	Anti-NMDAR-E (11)	EVE (16)	Control (20)
Gender, female/male	4/12	5/6	5/11	9F, 11M
Age, median (range), y	6.3 (2–13.3)	6 (2.6–14)	2.7(1.3–9.5) *	4.9 (0.3–14)^#^
ICU admission (n), duration (d)	2/16 (12%) (3–4 d)	4/11 (36%) (21–26 d)	7/16 (44%) (1–7 d)	NA
Timing of CSF from onset of acute neurological symptoms, median (range), days	4 (1–21)	16 (4–24) *	3 (1–12)	NA
CSF Pleocytosis (>5x10^6^ cells/L)	13 (81%)	8 (72%)	11 (68%)	0
CSF Pleocytosis, median (range), x10^6^ /L	33 (0–175)	15 (0–170)	43.5 (0–86)	0 (0–3)
CSF neutrophils, median (range), x10^6^ /L	**4 (0–25)**	0 (0–3)	2 (0–10)	0 (0–1)
CSF protein, median (range), mg/dl	0.36 (0.15–0.7)	0.22 (0.1–0.77)	0.26 (0.1–0.31)	NA
Neopterin (Elevated> 29 nmol/L), n	13 (81%)	11(100%)	16 (100%)	1(6PTPS deficiency)
Oligoclonal IgG bands (positive), n	2/15 (13%)	4/11 (36%)	1/10 (10%)	NA
MRI Brain abnormal, n	16/16 (100%)	3/11 (27%)	14/15 (93%)	-
MRI spine abnormal	9/12 (75%)	Not done	7/10 (70%)	
Immunotherapy	15/16 (93%) [Steroids (n = 15), IVIG (n = 1), mycophenolate (n = 2)]	10/11 (90%) [Steroids (n = 10), IVIG (n = 8), rituximab (n = 3)]	8/16 (50%) [Steroids (n = 6), IVIG (n = 6)	-
Relapse	3/16 (18%)	3/11(27%)	0/16 (0%)	-
Duration of FU, median (range), mo	16 (1–54)	**27 (11–66)**	6 (1–30)	-
FU outcome (MRS score)				
No disability (0–1)	10/16 (62%)	6/11 (54%)	11/16 (68%)	** **
Mild disability (2)	4/16 (25%)	2/11 (18%)	1/16 (6%)	
Moderate disability (3)	2/16 (12%)	3/11 (27%)	1/16 (6%)	

*Highlighted results represent variables with P<0.05 in the Kruskal Wallis test, see results section for more details

^#^ age at the time of CSF sampling

**Abbreviations:** ADEM, acute disseminated encephalomyelitis; Anti-NMDAR E, anti-N-Methyl D-Aspartate receptor encephalitis; EVE, entero viral encephalitis; y, years; d, day; mo, months: CSF, cerebrospinal fluid; MRI, magnetic resonance imaging; ICU, intensive care unit admission; MRS, modified Rankin scale; FU, follow up; IVIG, immunoglobulins

Twenty non-inflammatory neurological controls (Table 1) were used to generate a reference range, and included cerebral palsy (n = 8) [kernicterus (2), extreme prematurity (1), placental insufficiency (1), unknown cause (4)], neurotransmitter disorders (n = 4) [dopa responsive dystonia (3), and 6PTPS deficiency (1)], monogenic movement disorders (n = 6), stereotypy (n = 1), and congenital myasthenic syndrome (n = 1).

### Multiplex cytokine/chemokine immunoassay

Thirty-two cytokines were measured by multiplexed fluorescent bead-based immunoassay detection (MILLIPLEX® MAP system, Millipore Corporation, Missouri U.S.A.) according to the manufacturer’s instructions, using a combination of 23-plex (MPHCYTOMAG60K23), 6 plex (MPHCYP2MAG62K06), and 3-plex (MPHCYP3MAG63K03) Millipore Human Cytokine panel kits. The 23-plex kit contained antibody-conjugated beads for following cytokines and chemokines: IL-1ra, GM-CSF, IL-1b, TNF-α, IL-2, IL-4, IL-6, IL-8, IL-10, IL-13, IL-17A, IFN-γ, CCL2/MCP-1, CCL5/RANTES, CXCL1/GRO, CXCL10/IP-10, CCL3/MIP-1a, CCL4/MIP-1b, IL-12 (p40) and IL-12 (p70), IFN-α, G-CSF and CCL11/Eotaxin. The 6-plex kit was used to detect IL-21, IL-23, CXCL13/BCA-1, CCL17/TARC, CCL21/6Ckine and CXCL12/SDF-1. The 3 Plex kit contained antibody-conjugated beads for CXCL9/MIG, CXCL11/I-TAC, and CCL19/MIP-3b. All CSF samples were primarily obtained for routine diagnostic work-up prior to commencing treatment, and samples were frozen at -40°C till use for the analysis. The CSF samples were thawed overnight for analysis. For each assay, the curve was derived from various concentrations of the cytokine standards assayed in the same manner as patient samples. All samples were measured undiluted.

### Enzyme-linked immunosorbent assay (ELISA)

APRIL (sensitivity >2pg/ml) and BAFF (sensitivity >0.4ng/ml) were performed using ELISA kits as per the manufacturer’s instructions (R&D Systems, Minneapolis, MN). All samples were analyzed undiluted except 9 samples, where volume was insufficient for BAFF analysis. In these cases, results were multiplied by the appropriate dilution factor.

The methodological details including assay method, lower detection limits and coefficient of variance are available at the manufacturer’s website, http://www.merckmillipore.com. The detectability of cytokine/chemokines was variable in control groups and up to 40% of cytokines and chemokines (n = 14/34) were above detection limit in ≥50% controls as shown in Table A in S1 File.

### Statistical analysis

Statistical analysis was performed using SAS version 9.3 and *R*: *A language and environment for statistical computing* programme v 3.2.2. The graphs were composed using Graph Pad Prism software version 6. In order to assess the clinical utility of the CSF cytokine/chemokines as biomarkers to diagnose all encephalitis patients (ADEM, anti-NMDAR E and EVE), we calculated sensitivity using 95^th^ centile of control value as the upper limit of normal range. CSF cytokine/chemokines were also transformed according to log10 (biomarker+(minimum non-zero biomarker value)/2) and tested in univariable logistic regression models to predict inflammation in encephalitis versus controls. The cytokine/chemokines with area under the receiver operating characteristic (ROC) curve (AUC) of at least 0.8 were then tested in a multivariable model with backward selection.

Statistical analyses of nonparametric continuous data (clinical parameters and cytokine/chemokine levels in encephalitis groups) were performed using Kruskal-Wallis test for multiple groups with Bonferroni correction and 2-tailed P values were calculated. We also compared cytokine/chemokines concentrations in all encephalitis patients categorised based on severity of disease at nadir (MRS = 5, severe and MRS<5, less severe) or presence of disability at final follow-up (MRS 0 or 1, no disability, and MRS 2 or more, disability present) using the Mann-Whitney U test.

The Spearman rank correlation coefficient was used for analyses of correlations between cytokine/chemokines, and between cytokine levels and clinical parameters in the encephalitis groups. One-way Hierarchical clustering was performed using Spearman’s correlation coefficient as a proximity distance matrix, which was then plotted using a dendrogram. Heat map was created for each of the diagnostic groups using the “heatmap.2” function in *R*. The cytokine/chemokine molecules are listed in the same order for each of the three encephalitis groups to allow a visual comparison of the pattern between the groups. The heat maps colors correspond to correlations grading from -1 (negative correlation, red), no correlation (white) to 1 (positive correlation, black). Similarly heat maps were also generated for all encephalitis patients based on severity of encephalitis at nadir, and final follow up outcome using MRS score as described earlier.

## Results

### Comparison of clinical features and investigations between ADEM, anti-NMDAR-E, and EVE groups (Table 1)

The children with ADEM and anti-NMDAR-E were older compared to EVE groups (p = 0.01). The CSF timing from onset of neurological symptoms was shorter in ADEM and EVE group compared to anti-NMDAR-E (p<0.001, ADEM vs anti-NMDAR-E; p<0.001, anti-NMDAR-E vs EVE). The sensitivity of CSF pleocytosis (68–81%), neopterin (81–100%) and oligoclonal bands (OCB, analysed using isoelectric focusing) (10–36%) to detect inflammation in individual encephalitis groups is shown in Table 1. CSF neutrophils were higher in ADEM group (p = 0.01, anti-NMDAR-E vs ADEM). CSF protein was higher in ADEM and EVE compared to anti-NMDAR-E. There was laboratory evidence of preceding infection in five patients with ADEM as shown by positive serum serology (EBV = 2, Mycoplasma = 2) or isolation of H1N1 virus on nasopharyngeal aspirate (n = 1). The follow-up period was longer in ADEM and anti-NMDAR-E compared to EVE group (anti-NMDAR-E vs EVE, p = 0.004). Recurrent clinical relapses occurred in 3/16 of ADEM patients 2 months, 8 months and 3 years after the initial events (1–3 episodes) whereas 3/11 patients with anti-NMDAR E had relapses 4, 8 and 18 months after the initial illness (1–5 episodes). There were no relapses in EVE. The details of the immunotherapy administered are presented in Table 1. The children with ADEM (n = 6/16) and anti-NMDAR-E (n = 5/11) were more likely to report symptoms related to cognitive deficit on follow up than EVE (n = 2/16).

### Sensitivity and specificity of cytokine/chemokines in encephalitis groups (Table 2, Fig 1)

**Fig 1:**
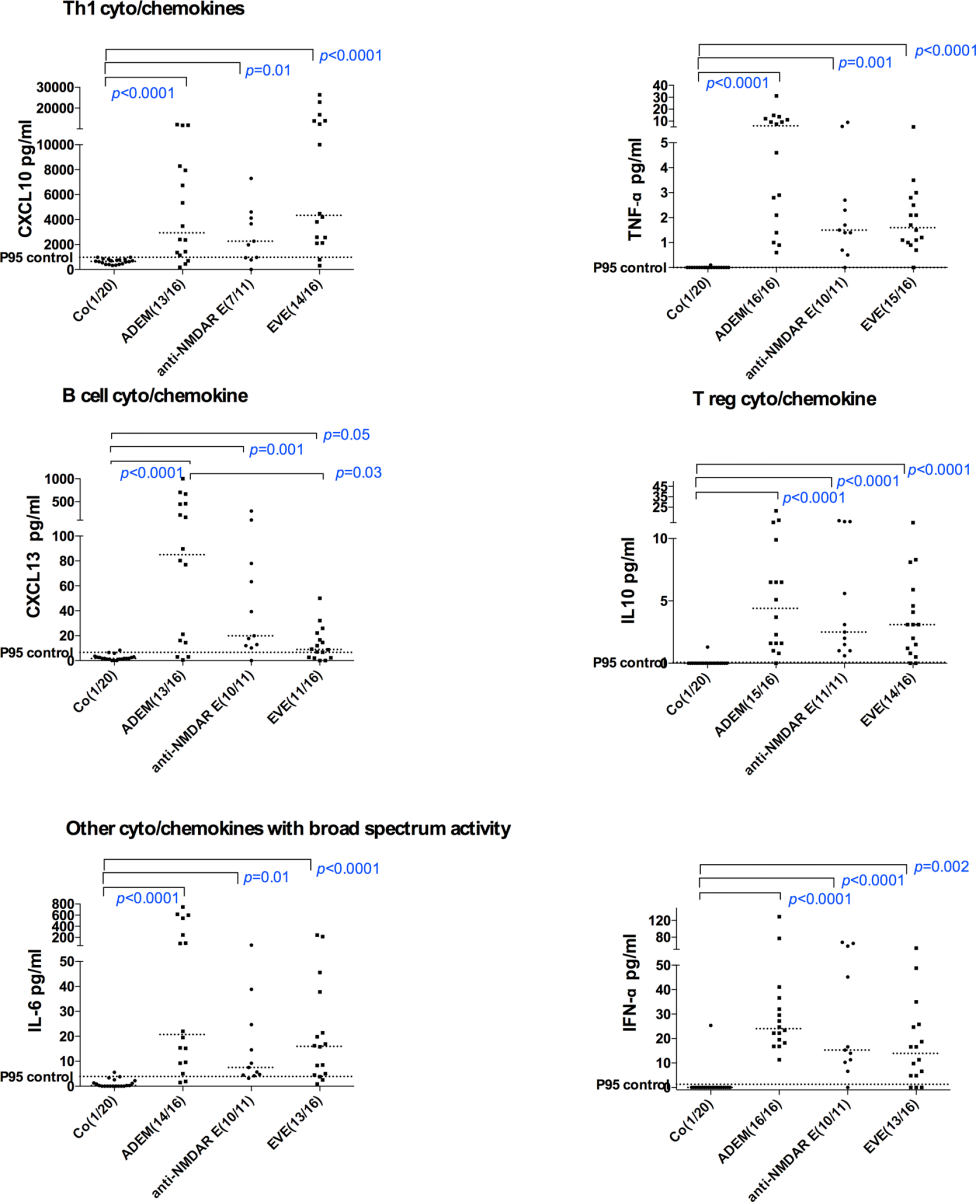
CSF concentrations of cytokine/chemokines with >75% sensitivity to detect intrathecal inflammation in all children with acute encephalitis- acute disseminated encephalomyelitis (ADEM), anti-NMDAR encephalitis (anti-NMDAR E) and enteroviral encephalitis (EVE). Dotted lines represent medians. The statistical analysis was performed using Kruskal Wallis test. The 95% centile of the control values are presented (P95 control).

We observed that TNF-α, IL-10, IFN-α, IL-6, CXCL10 and CXCL13 demonstrated >79.1% sensitivity in patients with encephalitis using control 95^th^ centile as cut off (specificity 95%). These cytokine/chemokines are presented according to Th or B cell status in Fig 1. All molecules detected in more than 50% of encephalitis patients are presented in descending order of sensitivity, and are listed in Table 2. Other molecules including Th1 (IFN-γ,), Th2 (IL-2. IL-4 CCL11, CCL17, CCL21), Th17 (IL-17A, GM-CSF), B cell molecules (BAFF, APRIL) and other cytokine/chemokines including IL-21, IL-1b, IL-12p40, CCL2 and IL-12p70 were detected in less than 50% of patients with encephalitis. On univariable regression analysis, thirteen cytokine/chemokines had AUC of >0.8 (from highest to lowest AUC: TNF-α, IL-6, IL-10, IFN-α, IL1ra, CXCL10, CXCL13, CXCL9, G-CSF, IL-13, RANTES, IL-12p40, CCL19). On multiple logistic regression analysis, the combination of IFN-α and IL-6 together fitted the model best of all and remained after backward selection with AUC 0.99 (CI 0.97–1.00) [odds ratio (OR) for log (IL-6log): 122 (CI 1.9->999), P 0.024; OR for log (IFN-α) log: 124 (CI 3.5->999), P 0.008] to predict inflammation in encephalitis. CSF concentrations of CXCL13, CXCL12 (low), CXCL10 and IFN-α were also elevated in clinical relapses of two cases with anti-NMDAR E (data not shown).

**Table 2:** The sensitivity of CSF cytokine/chemokines in ADEM, anti-NMDAR E, EVE and all encephalitis patients compared to controls. The molecules are presented in descending order of sensitivity in all encephalitis groups.

Cytokine/ chemokines	95th centile control concentration (pg/L) (95% specificity)	Number of patients with elevated cytokine/chemokines above 95th centile control value (Sensitivity)			
		ADEM (16)	Anti-NMDAR-E (11)	EVE (16)	All E (43)
**TNF-α**	0	16 (100%)	10 (90.9%)	15 (93%)	41(95.3%)
**IL-10**	0.1	15 (93%)	11 (100%)	14 (87.5%)	40 (93%)
**IFN-α**	1.3	16 (100%)	10 (90.9%)	13 (81%)	39 (90.7%)
**IL-6**	3.9	14 (87.5%)	10 (90.9%)	13 (81%)	37 (86%)
**CXCL13**	6.6	13 (81%)	10 (90.9%)	11 (68.7%)	34 (79.1%)
**CXCL10**	976.4	13 (81%)	7 (63.6%)	14 (87.5%)	34 (79.1%)
**IL-1ra**	9.4	13 (81%)	7 (63.6%)	12 (75%)	32 (74.4%)
**CXCL9**	72	14 (87.5%)	6 (54.5%)	12 (75%)	32 (74.4%)
**IL-13**	0	15 (93%)	7 (63.6%)	9 (56.3%)	31 (72.1%)
**G-CSF**	32.7	12 (75%)	6 (54.5%)	13 (81%)	31 (72.1%)
**RANTES**	105.4	14 (87.5%)	4 (36.4%)	11 (68.7%)	29 (67.4%)
**IL-8**	50.2	13 (81%)	5 (45.4%)	10 (62.5%)	28 (65.1%)
**IL-23**	0	12 (75%)	6 (54.5%)	7 (43.8%)	25 (58.1%)
**CCL19**	219.8	13 (81%)	3 (27.3%)	8 (50%)	24 (55.8%)
**CXCL1**	11.1	11 (68.7%)	5 (45.4%)	7 (43.8%)	23 (53.5%)
**CCL3**	15.7	12 (75%)	6 (54.5%)	4 (25%)	22 (51.2%)
**CXCL12**	1145**(n< 5**^**th**^ **centile)**	5 (31.3%)	7 (63.6%)	10 (62.5%)	22 (51.2%)

**Abbreviations:** E, encephalitis, TNF, Tumor necrosis factor; IL-, interleukin; IFN-, Interferon; G-CSF, granulocyte colony—stimulating factor; RANTES, Regulated on activation normal T cell expressed and secreted

### Comparison of CSF cytokine/chemokine levels between ADEM, anti-NMDAR E, EVE, and controls (Fig 2)

**Fig 2:**
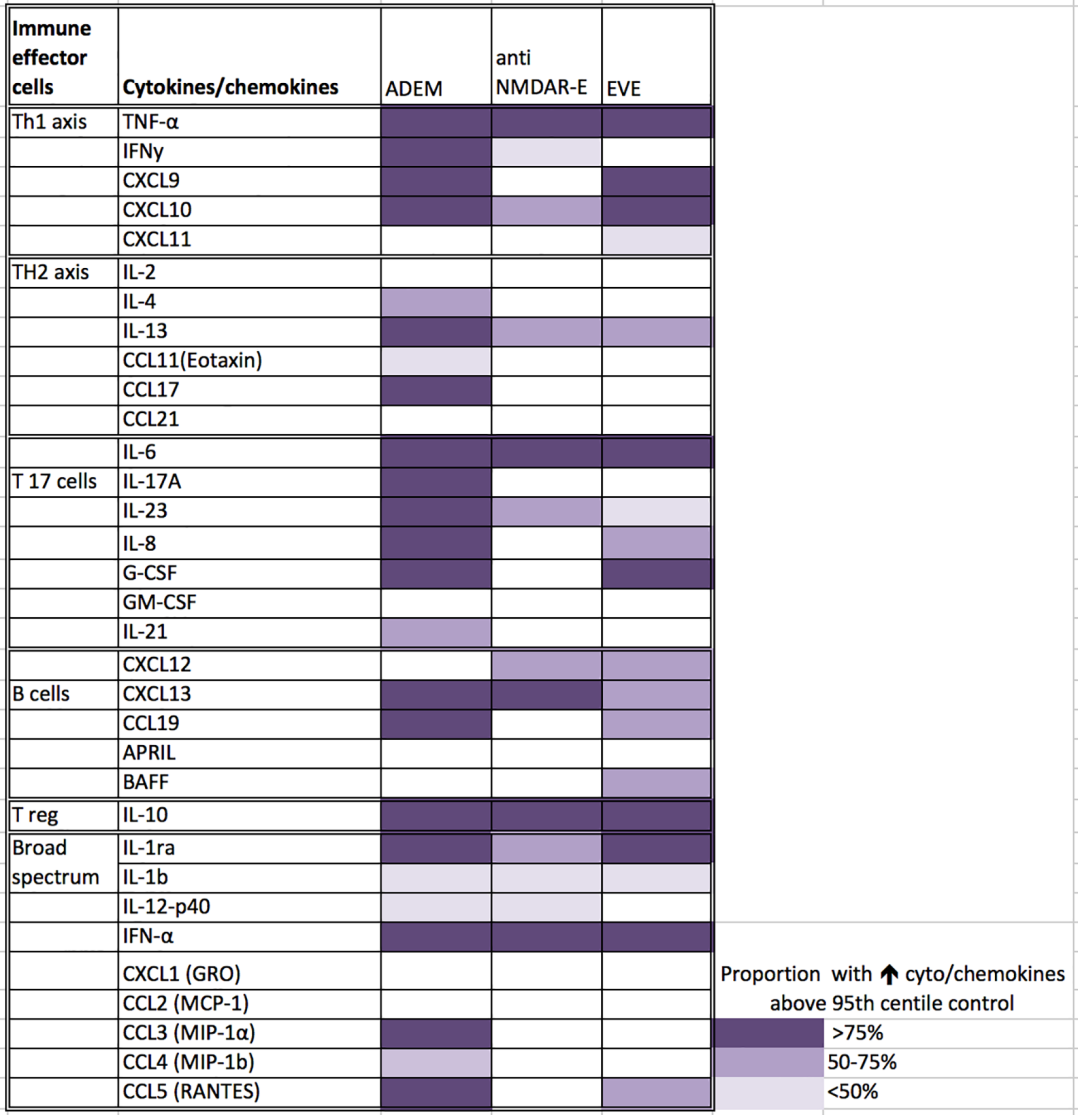
Heat map of elevated CSF cytokine/ chemokines^#^ in encephalitis groups compared to controls presented according to T and B cell subsets. There is a broad elevation of cytokine/chemokines related to all Th helper subsets (Th1, Th2, T reg, Th17, B cell and other cytokines and chemokines) in ADEM patients unlike anti-NMDAR E and EVE. ^#^ Cytokine/chemokines were shaded only if these molecules were statistically significantly elevated in different encephalitis groups compared to controls (p<0.05).

A heat map was generated to demonstrate statistically significantly elevated cytokine/chemokines and their frequency of elevation in different encephalitis groups compared to controls (Fig 2). A broad range of cytokine/chemokines were elevated in ADEM compared to anti-NMDAR E and EVE groups, and in addition the ADEM group showed more common involvement of Th2 (IL-4, IL-13, CCL11, CCL21) and Th17 (IL-17A, IL-23, IL-8, G-CSF, IL-21) cytokines compared to controls (S1 File: Table B and S1 Fig). The patients with MOG antibody positive associated demyelination showed predominant elevation of B cell related cytokine/chemokines (CXCL13, APRIL, BAFF and CCL19) compared to MOG antibody negative demyelination group [15] as well as other encephalitis groups (CXCL13 and CCL19) (S1 Fig).

**Fig 3:**
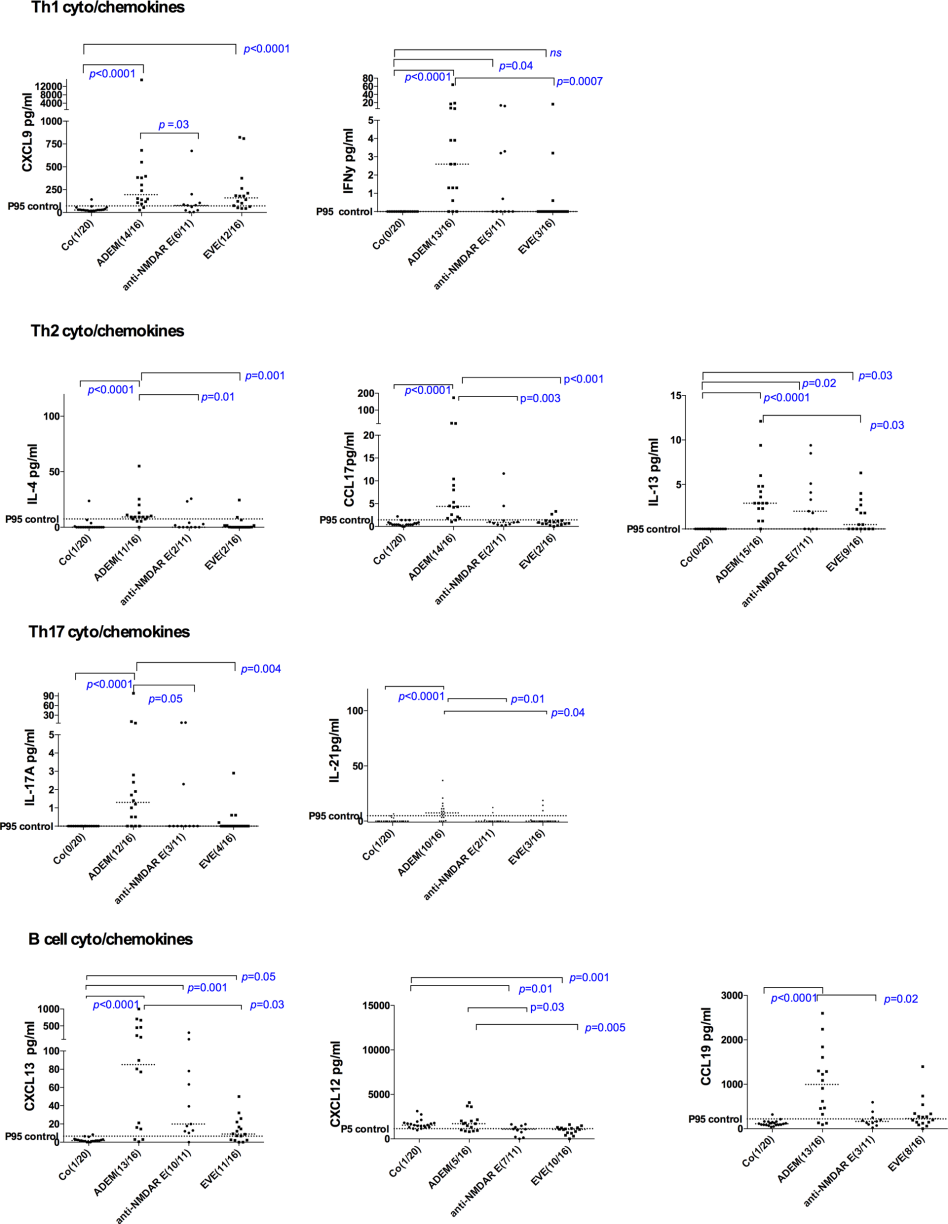
CSF cytokine/chemokine levels that were more elevated in ADEM compared to EVE and anti-NMDAR E. Th17 (IL-21, IL-17A) and Th2 (CCL17, and IL-4) related cytokine/chemokines showed statistically significant elevation in ADEM compared to both EVE and anti-NMDAR E. CXCL12 showed paradoxical decrease in EVE and anti-NMDAR E, in contrast to elevation noted in ADEM.

There were no significant differences in cytokine/chemokine concentrations between EVE and anti-NMDAR E groups. Th1 (CXCL9, IFN -γ), Th2 (CCL17, IL-4 and IL-13), Th17 (IL-21, IL-17A) and B cell (CCL19, CXCL13) related cytokine/chemokines showed statistically significant elevation in ADEM compared to EVE and/or anti-NMDAR E (Fig 3). CXCL12 showed paradoxical decrease in EVE and anti-NMDAR E, in contrast to elevation noted in ADEM.

### Correlation between CSF cytokine/chemokines and clinical parameters in patients with ADEM /anti-NMDAR E/ EVE

We performed comparisons between most sensitive CSF cytokine/chemokines (CXCL13, CXCL10, IFN-α, IL-10, IL-6 and TNF-α) and CSF protein, CSF pleocytosis, timing of CSF sample from the onset of neurological symptoms, and age at encephalitis presentation using spearman correlation tests (non parametric test, n = 24 comparisons were made in each analysis group). The majority of the correlations between CSF protein and CSF cytokines were not significant except for moderate correlation with CXCL13 (r = 0.53, P = 0.04), IL-10 (r = 0.69, P = 0.003) and TNF-α (r = 0.63, P = 0.009) in ADEM, and IL-10 in EVE (r = 0.67, P = 0.007). Only CSF CXCL13 showed a positive correlation with CSF cell count in all encephalitis patients (r = 0.54, P = 0.0002). CSF cytokines/chemokine levels generally declined with time of CSF sampling although the correlations were not significant in EVE, Anti-NMDAR E, ADEM and all encephalitis groups. There was no correlation between age and CSF cytokine and chemokine levels in different encephalitis groups. There was no evidence of correlations between CSF cytokine/chemokines and severity of disease at presentation or disability outcome using modified Rankin scale (data not shown). There was no evidence of differences in cyto/chemokine concentrations in all encephalitis patients grouped according to severity of the disease or presence of disability.

### Cluster analysis of cytokine/chemokine correlations in different encephalitis groups and outcome groups (Figs 4 & 5)

Hierarchical clustering was used to identify sets of cytokines whose expression levels among patients within individual disease category are correlated. The results are represented in heat maps to facilitate visualization of interaction between cytokine/chemokines among a large set of variables in individual disease groups and are unaffected by heterogeneity in overall cytokine levels within each group. The cytokines and chemokine formed two major clusters of cytokines and chemokines, which correlated within each group. First cluster (Cluster A) included Th1 (TNF-α, IFN-γ), Th2 (IL-2, IL-4, IL-13), Treg (IL-10) and Th17 (IL-17A, IL-21, G-MCSF, G-CSF, IL-6, IL-8, IL-23) and other molecules (IL-1b, IL-1ra, IFN- α). The second cluster (Cluster B) molecules included Th1 (CXCL9, 10 &11), B cell related chemokines (CCL19, CXCL12, CXCL13, APRIL) and other molecules (RANTES, CCL17). The clustering pattern showed some similarities in both anti-NMDAR E and ADEM groups and showed positive correlations within cluster A molecules. However, in contrast to ADEM, there were significant negative correlations between cluster B (CXCL12, CXCL9 and 10) and cluster A molecules (shown in red) in anti-NMDAR E. The correlations between the molecules were weaker in EVE compared to anti-NMDAR E and ADEM. As shown in Fig 5, the cluster B molecules showed similar higher correlations between those with higher severity of encephalopathy and those with disability in follow up in all encephalitis patients. The cytokine pattern also showed high positive correlation between cluster A and B in all encephalitis patients with disability compared to those with no or mild disability which may therefore indicate a cooperative function (data not shown).

**Fig 4:**
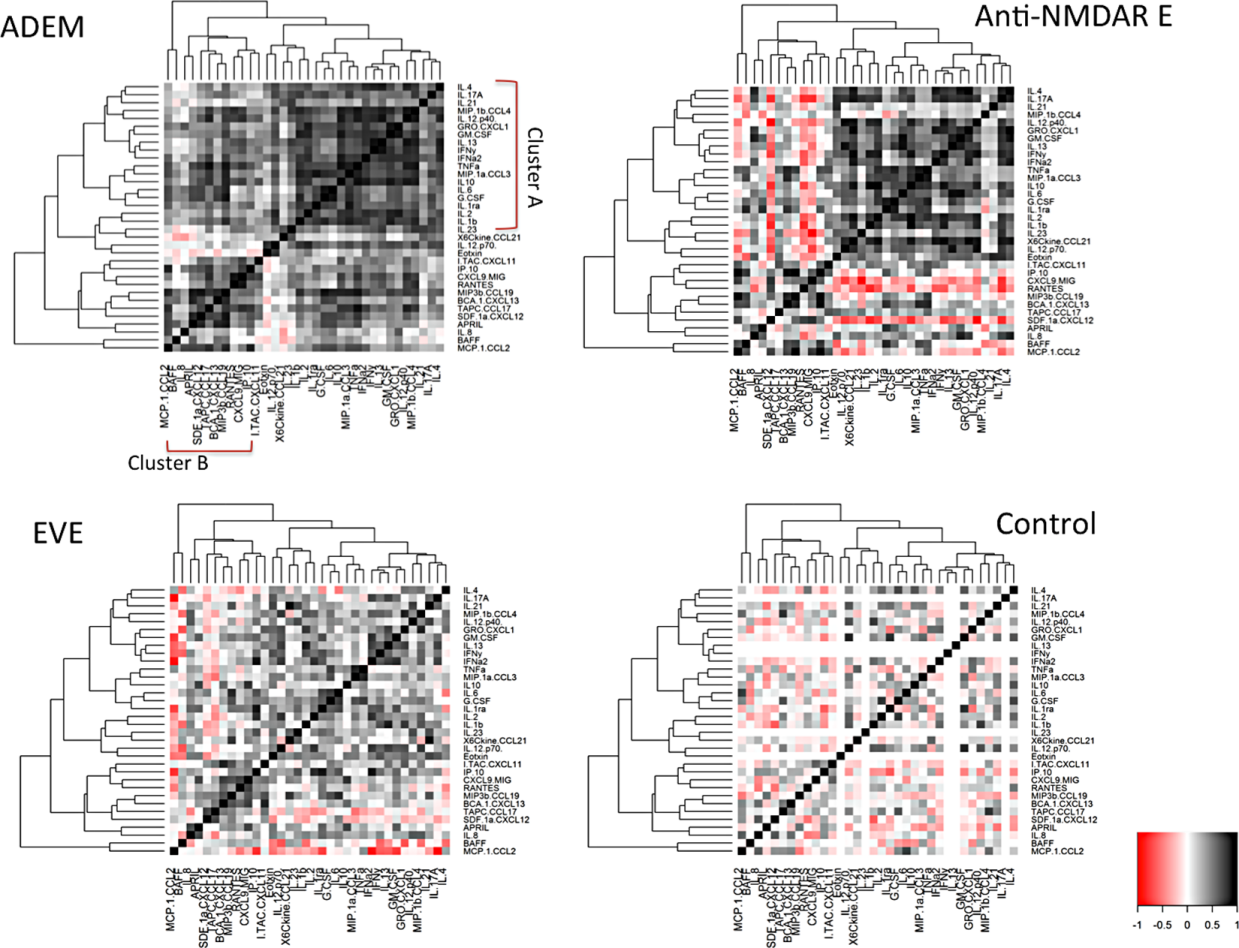
Hierarchical cluster analysis heat-map showing nearest-neighbour correlations of cytokines and chemokines in ADEM, anti-NMDAR E, EVE and controls. Cytokines with positive correlations are represented in graded shades of black and negative correlations in graded shades of red. The same order of the analytes along axis is used for all the three heatmaps to allow comparisons. The clustering pattern showed some similarities (cluster A) in immune mediated encephalitis (ADEM and anti-NMDAR E), which was less observed in viral encephalitis and controls.

**Fig 5:**
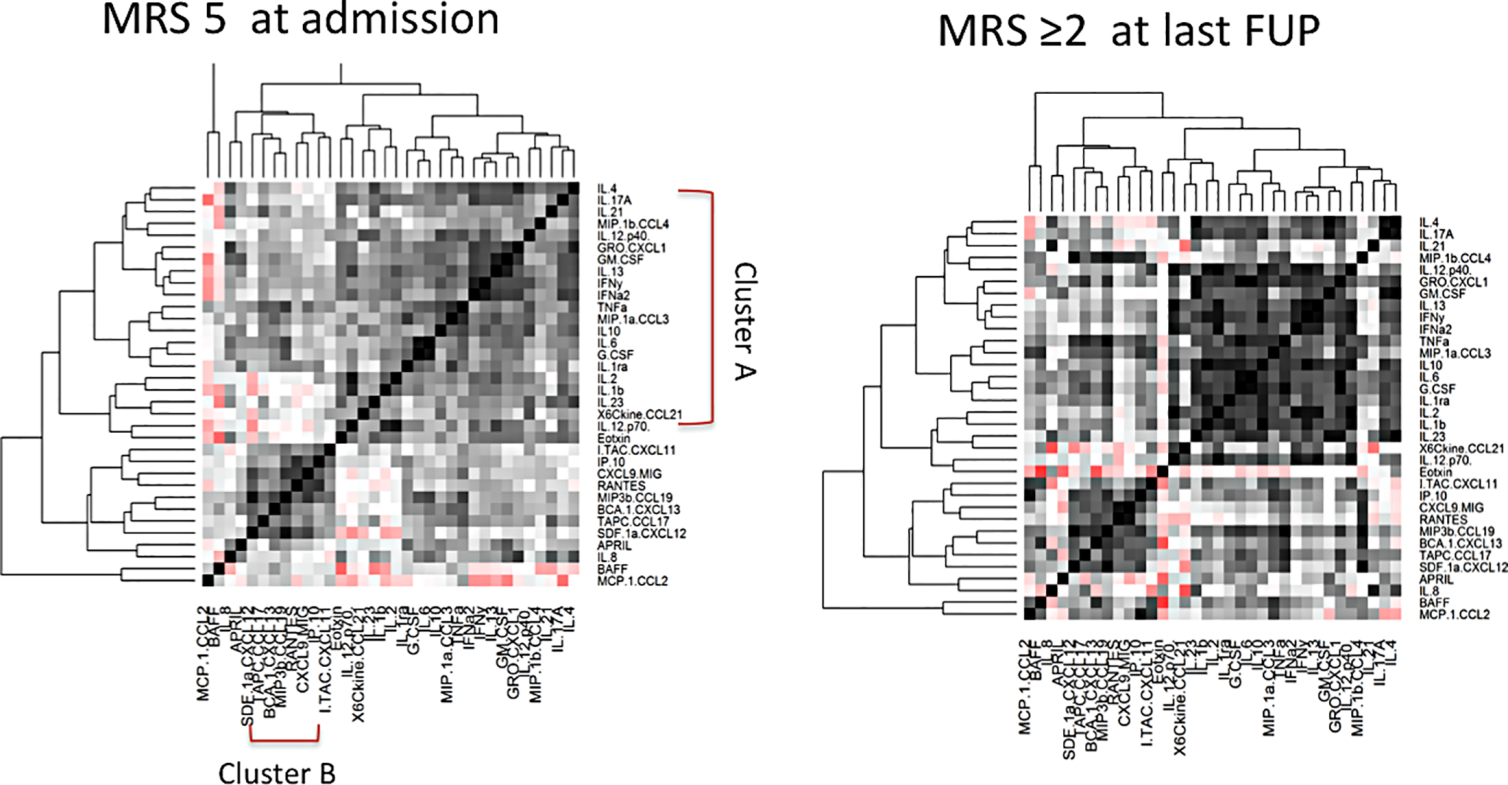
Heat map representation of cytokine/chemokine molecule interaction in the CSF of patients with all encephalitis with severe encephalopathy at admission (modified Rankin scale, MRS 5) and worse disability at follow up (MRS >2). The cluster B molecules showed similar positive correlations between those with higher severity of encephalopathy and those with disability in follow up.

## Discussion

The aim of the present study was to describe the CSF cytokine/chemokine concentrations in patients in well-defined encephalitis groups and discuss their utility as biomarkers of inflammation. This is based on the hypothesis that CSF cytokines may correspond to the intrathecal activation of immunoactive cells, and the evaluation of these cytokines might be a good indicator of the type of immune activation, the severity of inflammation and disease activity in neuroinflammatory disorders of the brain. In our cohort, among currently available investigations, the sensitivity of pleocytosis, oligoclonal bands and neopterin were similar to previous studies [4, 7, 9, 18, 19]. CSF neopterin performed the best of these commonly used CSF biomarkers, although is not used commonly in neurology practice outside of HIV neurology [20]. These findings highlight the need to develop more diagnostic markers, which can guide clinicians in diagnosing, monitoring and treating neuroinflammation.

In our cohort of encephalitis, the Th1 (CXCL10 & TNF-α), T reg (IL-10), B cell (CXCL13) related cytokine/chemokines, and cytokines with broad spectrum of activities including IL-6 and IFN-α, were elevated in more than 75% of the cases. The combination of IL-6 and IFN-α predicted inflammation on multiple regression analysis. In our study, we used non-inflammatory neurological controls to generate a reference range. The ‘normal cut off’ for CXCL13, CXCL10 and IFN-α in our study was similar to normal reference values reported in the literature [1, 21]. The ‘normal cut-off’ in our current study for TNF-α, IL-10 and IL-6 were lower than previous studies [1, 22], and the sensitivity for TNF-α, IL-6 and IL-10 molecules dropped when we used previously proposed reference values of cytokine/chemokine concentrations from the literature (48% for TNF-α when 2.1 pg/dl was used as cutoff, 72% for IL-6 when 6.2 pg/dl was used as cut off, and 37% for IL-10 when 5.3pg/dl was used as cut off) [1]. It should therefore be emphasized that the type of control group or reference range will influence the findings.

CSF CXCL13, CXCL10, IL-6 and IFN-α are elevated in a number of infectious and immune disorders. The cellular source, cellular target, role in neuroinflammation, and association with neuroinflammatory disorders and other neuroinflammatory markers are presented in the S1 File: Table C. Bielekova et al reported the utility of IL-12p40, CXCL13 and IL-8 as markers of inflammation based on CSF cytokine/chemokine studies in adult patients with multiple sclerosis [23] whereas Kowarik et al reported CXCL13 as a major determinant of inflammation using infectious and autoimmune inflammatory neurological disorders [24]. CXCL13 has been shown to be useful to monitor clinical progression and response to treatment in particularly in Lyme disease, anti-NMDAR encephalitis, multiple sclerosis and B cell lymphoproliferative disorders [21, 25–28]. Some reported variability in utility of cytokines and chemokines as biomarkers may be related to differences in case selection, CSF sampling time, disease categories, acuity of the illness and nature of the controls in addition to pleiotropic nature of these molecules. Due to these variables, a combination of cytokine/chemokines may be more discriminating than a single parameter. Of 3 cases studied with encephalitis relapses, IFN-α was elevated all cases where as CXCL10 and CXCL13 were elevated only in two cases.

When we compared the cytokine/chemokine profile in different encephalitis groups, patients with ADEM showed predominant elevation of Th1 (IFN-γ, TNF-α, CXCL9, CXCL10), Th2 (IL-4, Eotaxin, CCL17, IL-13), Th17 (IL-23, G-CSF, IL-6, IL-8 and IL-17A), B cell (CXCL13, BAFF, CCL19) and other cytokines (CXCL1, IFN-α 2, IL-1ra) molecules, which supports the hypothesis that both cell mediated and humoral effector mechanisms may play a role in this condition, similar to experimental allergic encephalomyelitis (EAE) model in mouse [29, 30]. In a study of 14 children with ADEM, Ishizu et al showed elevation of cytokines related to the activation of macrophages/microglia and Th1 and Th2 cells except for IL-17 [31]. In contrast to this study, IL-17 was elevated in our ADEM cohort. CSF IL-17 acts as a potent inflammatory mediator by inducing cytokines and promoting monocyte and neutrophil recruitment to the inflammation site, and is elevated in patients with multiple sclerosis and Neuromyelitis optica [32, 33] [34] [35]. We have previously shown that patients with anti-myelin oligodendrocyte (MOG) antibodies have more elevated concentrations of B cell related and some of Th17 (G-CSF & IL-6) related cytokines compared to MOG antibody negative demyelination [15]. Therefore even though we have stringently selected our groups to be as homogenous as possible, there are likely subgroups within these groups that could influence findings.

The elevation of cytokine/chemokines in EVE is consistent with previously reported studies and the elevation of CXCL13, BAFF and APRIL highlight humoral mechanisms involved in protection against viral infections, possibly as a consequence of induction of these molecules by interferons [36–41]. The data on CSF cytokine and chemokine profile in autoimmune encephalitis is limited in the literature, and have shown elevation of CSF IL-6 and CXCL13 in previous studies [1, 21, 42]. Recently Liba et al analysed serial CSF cytokine/chemokines on 9 children and young adults with anti-NMDAR E and reported intrathecal elevation of CXCL10, CXCL13 during the acute phase of anti-NMDAR E whereas T cell molecules (INF-γ, TNF-α and IL-17A) were elevated in lower concentrations throughout the clinical course over months [43]. In contrast to this study, we found widespread elevation of Th1, B cell, Treg and other cytokine molecules in the acute phase of anti-NMDAR E. The broad elevation of cytokine/chemokines suggests multiple immune cell involvement despite the previous studies reporting prominent plasma cell infiltrates and IgG deposits, lack of cell-mediated or complement-mediated neuronal cell death reported on histopathology and low density of inflammatory cells [44–47].

Another distinctive feature in chemokine profile was lower levels of CSF CXCL12 in EVE and anti-NMDAR encephalitis compared to elevated levels in ADEM. The normal polarized expression of CXCL12 on the basolateral surface of the microvasculature was reported to shift to the luminal side during inflammation leading to low levels in CSF [48], [49]. However the reason for the observed discrepancies in CSF CXCL12 levels between ADEM and the other two groups of encephalitis (EVE and anti-NMDAR E) is not clear [50]. We did not see any correlation between CSF neopterin and IFN-γ or α (data not shown) in contrast to the previous hypothesis that IFN-γ and IFN-α induced neopterin synthesis [51, 52] suggesting the possibility of alternate immune mechanisms of neopterin production [53].

There was no evidence of differences in cytokine/chemokine concentration between EVE and anti-NMDAR E suggesting shared immunological pathways involving T cell, B cell and macrophages. Similar to our study, Michael et al showed no major differences (except for IL-8, IL1ra, MPO) in cytokine, chemokine and other inflammatory mediator concentration between heterogeneous groups of infectious and autoimmune encephalitis in adults [54]. Despite observed similarities in cytokine/chemokine concentrations between EVE and anti-NMDAR E, there were differences in clustering pattern of cytokine/chemokines between the two groups suggesting a disease specific change in regulation of cytokine/chemokines which may be of functional relevance *in vivo* [54]. The observed positive correlations between Th1, Th17 and other cytokines in animal models of immune mediated encephalitis highlight the importance of their interactions contributing to autoimmune pathogenesis. Using EAE models, Domingues et al have shown that both Th1 and Th17 lineages possess the ability to induce CNS autoimmunity but can function with complementary as well as differential pathogenic mechanisms [55]. Previous studies also reported that imbalance between Th1 and Th 17 effector populations determines the type and severity of subsequent inflammatory response and distinct clinical/histopathological phenotypes of the disease in autoimmune EAE models depending on the cytokines produced by the disease-inducing T cells [56–58].

In our study, the correlations of CSF cytokine/chemokines with severity at admission and disability at follow up were not statistically significant unlike previous studies [21, 59, 60]. However a subset of cytokines showed positive correlations on cluster analysis heat maps in patients with severe encephalopathy at admission, and disability at follow up, and these molecules (CXCL13 and CXCL10) were also identified as markers of inflammation in all encephalitis groups irrespective of the cause. The observed correlations suggest that these molecules may be considered as potential targets of immune therapy. Tacrolimus (FK506) and monoclonal antibodies against CXCL10 have been shown to reduce clinical symptoms and specifically reduce high levels of intrathecal CXCL10, thereby reducing T cell access to the CNS with interruption of the feedback loop in CNS inflammation [61, 62]. Similarly anti-CXCL13 antibody demonstrated efficacy in mouse models of autoimmunity including multiple sclerosis by reducing B cell infiltration and subsequent interactions with T cells [63–65].

The important limitation of the study is that even though we measured a broad array CSF cytokines and chemokines in all encephalitis groups, we could not perform comparisons between serum and CSF samples to due to the lack of availability of stored frozen serum samples. Previous cytokine/chemokines studies on neuroinflammatory disorders of brain showed higher CSF to serum ratios of these molecules and some of these molecules have low transfer rate across blood brain barrier [38] [24, 39, 66–68]. The presence of blood CSF barrier dysfunction is possible in some of our patients. Future studies measuring blood CSF barrier dysfunction (albumin quotient) and simultaneous serum and CSF cytokine/chemokines are needed to unravel the origin of CSF cytokine/chemokines and extent of leakage of these molecules through the blood CSF barrier. The other limitation is that the sample size is moderately small in each individual group of encephalitis. In the infective group, only enteroviral encephalitis was chosen, as it is a common cause of viral encephalitis. It would be useful to examine less common viral encephalitis in children, such as herpes simplex encephalitis [9]. The control reference value used might have influenced the analysis of the sensitivity. The patients with anti-NMDAR E had later CSF sampling time, which may have affected cyto/chemokine concentrations. The interpretation of CSF cytokine/chemokines requires understanding of kinetics of expression, timing of CSF sample in relation to the disease, interactions between cytokine/chemokines and their receptors and the stage and severity of the disease. A further limitation is that CSF cytokine/chemokines are not specific and can be elevated in number of non-inflammatory disorders of brain [1].

## Conclusion

To conclude, we presented cytokine/chemokine profile from relatively well-defined encephalitis groups. Our study suggests that combining several biomarkers will enhance our ability to diagnose and monitor CNS inflammation. Despite similarities between CSF cytokine/chemokine concentrations in EVE and anti NMDAR E, we identified differential cytokine/chemokine interactions on cluster analysis, which may play a role in autoimmune pathogenesis (ADEM and anti NMDAR E). Some of these molecules (CXCL13 and CXCL10) may represent potential therapeutic targets in view of their association with severity of encephalopathy at admission and worse disability at follow up in all encephalitis cases.

Future clinical studies are needed to determine the utility of combination cytokine panels in diagnosing the presence of active intrathecal inflammation and monitoring disease activity in guiding therapeutic decisions. In addition to characterising the shared cytokine/chemokine functional pathways, other mechanisms need further investigation such as possible disturbances of immune active and immune regulatory cells, their signalling pathways and other molecules involved in regulating chemotaxis and degranulation which may be responsible for predisposition to autoimmunity following viral encephalitis. The potential role of monoclonal antibodies against CXCL10, CXCL13 as therapeutic targets in encephalitis needs further investigation.

## Supporting Information

S1 Fig CSF cytokine and chemokine concentrations among ADEM^#^, anti NMDAR E and EVE groups according to T and B cell effector groups. ^#^MOG antibody positive demyelination is shaded blue and MOG negative is shaded black in ADEM group.
(TIFF)

S1 File **Table A.** The detectability of CSF cytokine/chemokines in controls. **Table B.** Comparison between median CSF cytokine and chemokine concentrations in ADEM, anti NMDAR E and EVE groups according to T and B cell effector groups.**Table C.** Key Cytokine/chemokines, their cellular source, cellular target, role in neuroinflammation, and association with neuroinflammatory disorders and other neuroinflammatory markers.
(DOCX)

## Abbreviations

MRI: Magnetic Resonance Imaging
ADEM: Acute Disseminated Encephalo Myelitis
EVE: Enteroviral Encephalitis
E: Encephalitis
NMDAR: N-Methyl D-Aspartate Receptor
anti-NMDAR E: anti-NMDA R encephalitis
CSF: Cerebrospinal Fluid
IL: Interleukin
IFN: Interferon
