# Supplementary material for: Utility of CSF Cytokine/Chemokines as Markers of Active Intrathecal Inflammation: Comparison of Demyelinating, Anti-NMDAR and Enteroviral Encephalitis
Source: PLoS One. 2016 Aug 30;11(8):e0161656. doi: 10.1371/journal.pone.0161656 (PMC5004915; doi:10.1371/journal.pone.0161656)
Supplement: S1 File — Table A. The detectability of CSF cytokine/chemokines in controls. Table B. Comparison between median CSF cytokine and chemokine concentrations in ADEM, anti NMDAR E and EVE groups according to T and B cell effector groups.Table C. Key Cytokine/chemokines, their cellular source, cellular target, role in neuroinflammation, and association with neuroinflammatory disorders and other neuroinflammatory markers. (DOCX) [file pone.0161656.s002.docx]

**Table A: The detectability of CSF cytokines/chemokines in controls**

| **Cytokine/ chemokine** | **Min detection limit (pg/ml)** | **95th centile control (pg/L)** | **Frequency of elevation above detection limits (range, pg/ml)** |
| --- | --- | --- | --- |
| TNF-α | 0.7 | 0 | 0/20 |
| IL-13 | 1.3 | 0 | 0/20 |
| IL-23 | 31.5 | 0 | 0/20 |
| IFNy | 0.8 | 0 | 0/20 |
| IL-17A | 0.7 | 0 | 0/20 |
| Eotaxin/CCL11 | 4 | 0 | 0/20 |
| GM-CSF | 7.5 | 2.86 | 0/20 |
| IL-21 | 6.8 | 4.8 | 0/20 |
| IL 10 | 1.1 | 0.1 | 1/20 (1.3) |
| IFN-α | 2.9 | 1.3 | 1/20 (25.4) |
| CCL 21 | 45.4 | 16.9 | 1/20 (64) |
| CXCL1 | 9.9 | 11.1 | 1/20 (97.4) |
| IL-1b | 0.8 | 1.4 | 2/20 (1.4-1.9) |
| IL-12(p70) | 0.6 | 5.1 | 2/20 (5.1) |
| IL-4 | 4.5 | 7.5 | 2/20 (6.6-23.7) |
| IL-1ra | 8.3 | 9.4 | 2/20 (8.8-20.1) |
| IL-12(p40) | 7.4 | 18 | 3/20 (12.6-26.1) |
| IL-2 | 1 | 2.52 | 7/20 (1.3-2.9) |
| IL-6 | 0.9 | 3.9 | 8/20 (1.1-5.6) |
| RANTES | 1.2 | 105.4 | 8/20 (5.3-1799.9) |
| CCL4 | 3 | 12.3 | 10/20 (3.5-17.3) |
| CCL3 | 2.9 | 15.7 | 10/20 (3.5-22.7) |
| CXCL13 | 1.3 | 6.6 | 13/20 (1.8-8.4) |
| CXCL9 | 19.2 | 72 | 16/20 (22.5-143.7) |
| CCL17 | 0.4 | 1.4 | 17/20 (0.4-2.2) |
| APRIL | 2 | 4.3 | 17/20 (2.2-5.2) |
| CXCL11 | 1.7 | 8.2 | 18/20 (3.5-8.2) |
| BAFF | 0.4 | 425.3 | 20/20 (142.03-646.96) |
| IL-8 | 0.4 | 50.2 | 20/20 (19.6-77.3) |
| CXCL10 | 8.6 | 976.4 | 20/20 (327.4-985.6) |
| CCL19 | 3.1 | 219.8 | 20/20 (37.7-320.6) |
| G-CSF | 1.8 | 32.7 | 20/20 (5.9-36.2) |
| CCL2 | 1.9 | 1602.6 | 20/20 (633-3238.2) |
| CXCL12 | 55.8 | 1145 | 20/20 (981.5-3117.4) |

**Table B: Comparison between median CSF cytokine and chemokine concentrations in ADEM, anti NMDAR E and EVE groups according to T and B cell effector groups**

| **Cytokines/chemokines (pg/ml)** | **ADEM** | **Anti-NMDAR E** | **EVE** | **Control** | **ADEM vs Anti-NMDAR E** | **ADEM vs Anti-NMDAR E** | **Anti-NMDAR E vs EVE** |
| --- | --- | --- | --- | --- | --- | --- | --- |
|  | **Median (range)** | | | | **P values** | | |
| **Th1** |  | | | | | | |
| IFN-γ | 2.6 (0-63.9) | 0 (0-13.1) | 0 (0-16.3) | 0 (0) | 0.18 | **<0.001** | 0.81 |
| TNF-α | 6 (0.6-31.1) | 1.5 (0-9) | 1.6 (0-5.2) | 0 (0-0.1) | 0.27 | 0.21 | 1.00 |
| CXCL9/MIG | 195.9 (25.7-15123.9) | 77.8 (4.3-674.6) | 160.5 (43.9-822.4) | 26.9 (13.4-143.7) | **0.03** | 1.00 | 0.12 |
| IP-10/CXCL10 | 2940.65 (181.9-12070.3) | 2275.8 (0-268417.3) | 4341.35 (310.4-26391.7) | 646.9 (327.4-985.6) | 1.00 | 0.80 | 0.56 |
| CXCL11 | 4.15 (0-114.7) | 5.9 (2.1-31) | 6.6 (2.1-12.3) | 5.1 (0.4-8.2) | 1.00 | 0.44 | 1.00 |
| **Th2** |  | | | | | | |
| IL-2 | 0.3 (0-17.1) | 0.3 (0-5.5) | 0.5 (0-4) | 0.8 (0-2.9) | 1.00 | 1.00 | 1.00 |
| IL-4 | 9.3 (0-55.1) | 0 (0-25.7) | 0.1 (0-24.5) | 0 (0-23.7) | **0.01** | **0.001** | 1.00 |
| IL-13 | 2.9 (0-12.1) | 2 (0-9.4) | 0.5 (0-6.3) | 0 (0) | 0.58 | **0.03** | 0.85 |
| CCL17 | 4.4 (1-173.3) | 0.9 (0.2-11.6) | 0.8 (0-3.3) | 0.4 (0-2.2) | **0.003** | **<0.001** | 1.00 |
| Eotaxin (CCL11) | 0 (0-122.3) | 0 (0-34.5) | 0 (0-30) | 0 (0) | 0.50 | 0.21 | 1.00 |
| **Th17** |  | | | | | | |
| IL-6 | 20.75 (1.5-745.4) | 7.5 (3.2-60.4) | 16 (0.9-241.8) | 0.3 (0-5.6) | 0.60 | 0.95 | 1.00 |
| G-CSF | 78.35 (1.7-7378.6) | 35.9 (9.4-130.6) | 67.05 (14.8-1085.8) | 15.4 (5.9-36.2) | 0.36 | 1.00 | 0.36 |
| GM-CSF | 1.25 (0-38) | 0.4 (0-6.8) | 0.2 (0-3.6) | 0.2 ((0-4.1)) | 0.23 | **0.02** | 1.00 |
| IL-8 | 160.25 (6.5-516.7) | 43.7 (15.1-335.4) | 59.7 (17.2-2165.6) | 32.3 (19.6-77.3) | 0.24 | 0.89 | 1.00 |
| IL-17A | 1.3 (0-98) | 0 (0-7) | 0 (0-2.9) | 0 (0) | **0.05** | **0.004** | 1.00 |
| IL-23 | 10.1 (0-2276.2) | 46.7 (0-408.1) | 0 (0-369.6) | 0 (0) | 1.00 | 0.50 | 1.00 |
| **B cell** |  | | | | | | |
| MIP3b/CCL19 | 996.85(92.3-2598.8) | 164.7 (24.8-595.6) | 229.75 (1.4-1397.2) | 111.95 (37.7-320.6) | **0.02** | 0.06 | 1.00 |
| APRIL | 4.7(1.4-9.2) | 3.4 (1.4-12.80) | 3.100 (1.4-15.80) | 2.9 (1.4-5.200) | 1.00 | 1.00 | 1.00 |
| BAFF | 348.6(0-3029) | 352.6(58.5- 1405) | 537.2(28.66-2878) | 233.7(142-647) | 1.00 | 1.00 | 1.00 |
| BCA.1/CXCL13 | 85 (0.5-1000) | 19.9 (0-298.5) | 8.95 (0-50) | 1.8(0-8.4) | 1.00 | 0.03 | 0.37 |
| CXCL12 | 1704.9 (806.1-4081.6) | 1100.4 (0-1644) | 1069.2 (0-1612.7) | 1547.8(981.5-3117.4) | **0.03** | **0.005** | 1.00 |
| **T reg** |  | | | | | | |
| IL-10 | 4.4 (0-21.5) | 2.5 (0.6-12.1) | 3.1 (0-10.2) | 0 (0-1.3) | 1.00 | 1.00 | 1.00 |
| **Broad spectrum** |  | | | | | | |
| X6Ckine/CCL21 | 0 (0-1457.4) | 0 (0-1131.8) | 0 (0-166) | 0(0-64) | 1.00 | 1.00 | 1.00 |
| IL-21 | 7.4 (0-36.8) | 0 (0-12.3) | 0 (0-18.7) | 0(0-6.5) | **0.01** | **0.004** | 1.00 |
| GRO/CXCL1 | 89.65 (0-793.1) | 3.2 (0-126.3) | 0 (0-216.9) | 0 (0-97.4) | 0.45 | 0.09 | 1.00 |
| IFN-α | 24.05 (11.3-128.7) | 15.3 (0-67.7) | 13.95 (0-53.9) | 0 (0-25.4) | 0.86 | 0.12 | 1.00 |
| IL-12 (p40) | 9.6 (2.3-148.9) | 14.2 (0-63) | 5.65 (0-28.5) | 0(0-26.1) | 1.00 | 0.16 | 0.32 |
| IL-12 (p70) | 0 (0-28.4) | 1.5 (0-32.4) | 0.35 (0-25) | 0.7 (0-5.1) | 0.19 | 0.87 | 1.00 |
| IL-1ra | 46.3 (3.8-481.3) | 33.2 (0.9-167.3) | 19.6 (1.7-675.1) | 3.3 (0-20.1) | 1.00 | 1.00 | 1.00 |
| IL-1β | 0.7 (0-20.7) | 0.9 (0-7.4) | 0.9 (0-2.4) | 0 (0-1.9) | 1.00 | 1.00 | 1.00 |
| MCP-1/CCL2 | 870.65 (37.5-1558.5) | 901.6 (113.2-3121.9) | 651.6 (173.1-9859.6) | 967.75 (633-3238.2) | 1.00 | 1.00 | 1.00 |
| MIP-1α /CCL3 | 26.65 (0-90.9) | 21.6 (0-55.2) | 5.9 (0-30.5) | 1.75 (0-22.7) | 0.94 | 0.07 | 0.89 |
| MIP-1β/CCL4 | 13.4 (2.7-53.4) | 10.1 (0-27.5) | 7 (0-24.4) | 1.75 (0-17.3) | 1.00 | 0.08 | 0.89 |
| RANTES | 24.75 (5.9-205.6) | 7.5 (3.9-122.4) | 21.5 (0-102.4) | 0 (0-1799.9) | 0.29 | 0.89 | 1.00 |

**Abbreviations:** Th, T helper cell; Treg, Regulatory T cells; IL-, interleukin; IFN-, interferon; TNF, tumor necrosis factor; MIG, monokine induced by IFN-γ; IP, IFN-γ inducible protein; G-CSF, granulocyte colony stimulating factor; MIP, macrophage inflammatory protein; GM-CSF, granulocyte monocyte colony stimulating factor; APRIL, A proliferation inducing ligand; BAFF, B cell activation factor; BCA-1, B cell attracting chemokine, GRO, melanoma growth-stimulating activity alpha; MCP, monocyte chemotactic protein; RANTES, regulated on activation normal T cell expressed and secreted.

P value was calculated using Kruskal-Wallis test. P-values for each biomarker were corrected for multiple comparisons between the three groups, however there was no additional adjustment for the comparisons of multiple biomarkers. **Highlighted** results represent variables with P<0.05.

**Table C: Key Cyto/chemokines, their cellular source, cellular target, role in neuroinflammation, and association with neuroinflammatory disorders and other neuroinflammatory markers.**

| **Cytokine/**  **chemokine** | **Cellular sources** | **Action of molecule** | **Elevated in following Neuroinflammatory disorders** | **Clinical and other biomarker associations or correlations** |
| --- | --- | --- | --- | --- |
| CXCL13 | Follicular dendritic cells, CD4+CD57+ T cells, macrophages, thymic epithelial cells | Acts on CXCR5 receptor and helps in recruitment, clonal selection and expansion of B cells, persistence of chronic lymphocytic infiltration and ectopic follicle formation in CNS | Anti-NMDAR E [1], opsoclonus myoclonus ataxia syndrome (OMAS) [2], neuromyelitis optica (NMO) [3, 4], optic neuritis [5], multiple sclerosis (MS) [6, 7], and lyme neuroborreliosis [8] | Other biomarkers: Intrathecal IgG, CSF pleocytosis, OCB, CSF plasma cells  Clinical: Marker of disease severity, outcome/relapses and treatment response in MS, Lyme disease, NMO, OMAS, Anti-NMDAR E, Clinical isolated syndrome [9], [10] [2, 7, 11, 12],  [2, 4], [1, 3, 4] |
| CXCL10 | Lymphocytes, neutrophils, monocytes, astrocytes, neurons, and microglia | Acts on CXCR3 receptor expressed highly on Th1 cells, CD8+ T cells and natural killer (NK) cells, helps with recruitment and in Th1 cell mediated immunity | EVE [13, 14], OMAS [15], NPSLE [16, 17], MS, ADEM[18], NMO [19], and optic neuritis [5] | Other biomarkers: Correlates with cell count in ADEM/NMO [18], and GFAP [20] in NMO  Clinical: correlated with disease activity in NPSLE[21], Variable response to treatment in OMAS[15] |
| IL-6 | Macrophages, dendritic cells, monocytes, T cells | Differentiation of naïve T cells towards proinflammatory Th17 cell lineage in the presence of TGF-β and IL-23, producing additional cytokines/chemokines, Differentiation of B into plasma cells and promotes immunoglobulin synthesis  Acute phase protein synthesis | Viral encephalitis [22, 23], OMAS [24], NPSLE [25], NMO [20], and ADEM [26]. | Other biomarkers: CSF IGG synthesis, OCB, pleocytosis in ADEM & NMO and neuronal and glial markers in NMO [26, 27], [28, 29]  Clinical: EDSS, relapse in NMO[20], prognosis in herpes encephalitis[23] |
| IFN-α | Leukocytes, dendritic cells | Regulates innate and adaptive immunity against viruses and induces positive feed forward loop in activation of T, B, NK and dendritic cells and production of cyto/chemokines | NPSLE [17, 21], Japanese encephalitis [30] and Aicardi-Goutieres syndrome[31] | Variable response to disease activity [32, 33] |
| TNF-α | Macrophages, dendritic cells, monocytes, T cells, natural killer cells | Increase blood brain barrier permeability, toxic to oligodendrocytes, production of other proinflammatory cytokines, induction of Th17 cells along with IL-1 and IL-6 | MS [34, 35], Herpes encephalitis [36], and ADEM [37] | Variable correlation with relapse and disability in MS[38-40] |
| IL-10 | T cells, B cells, Monocytes | Helps in immunoregulation and inhibiting proinflammatory cytokine synthesis, | ADEM [27, 41], viral encephalitis [22, 23], NMO [20], Sydenham’s chorea [42], and occasionally in NPSLE [43], MS [7] | Other biomarkers: CSF GFAP, CSF cell counts and anti-AQP4 antibody titers in NMO[20]  Clinical: Positive correlation with outcome in encephalitis  [44] |

**Abbreviations:** OMAS, opsoclonus myoclonus ataxia syndrome; NMO, neuromyelitis optica; MS, multiple sclerosis; NPSLE, neuropsychiatric lupus erythematosus; OCB, oligoclonal bands; EDSS, end stage disability score; GFAP, Glial fibrillary acid protein; CSF, cerebrospinal fluid; IgG, immunolobulins

References:

1. Leypoldt F, Hoftberger R, Titulaer MJ, Armangue T, Gresa-Arribas N, Jahn H, et al. Investigations on CXCL13 in Anti-N-Methyl-D-Aspartate Receptor Encephalitis: A Potential Biomarker of Treatment Response. JAMA Neurol. 2014.

2. Pranzatelli MR, Tate ED, McGee NR, Travelstead AL, Ransohoff RM, Ness JM, et al. Key role of CXCL13/CXCR5 axis for cerebrospinal fluid B cell recruitment in pediatric OMS. J Neuroimmunol. 2012;243(1-2):81-8.

3. Zhong X, Wang H, Dai Y, Wu A, Bao J, Xu W, et al. Cerebrospinal fluid levels of CXCL13 are elevated in neuromyelitis optica. J Neuroimmunol. 2011;240-241:104-8.

4. Alvarez E, Piccio L, Mikesell RJ, Klawiter EC, Parks BJ, Naismith RT, et al. CXCL13 is a biomarker of inflammation in multiple sclerosis, neuromyelitis optica, and other neurological conditions. Mult Scler. 2013;19(9):1204-8.

5. Modvig S, Degn M, Horwitz H, Cramer SP, Larsson HB, Wanscher B, et al. Relationship between cerebrospinal fluid biomarkers for inflammation, demyelination and neurodegeneration in acute optic neuritis. PLoS One. 2013;8(10):e77163.

6. Khademi M, Kockum I, Andersson ML, Iacobaeus E, Brundin L, Sellebjerg F, et al. Cerebrospinal fluid CXCL13 in multiple sclerosis: a suggestive prognostic marker for the disease course. Mult Scler. 2011;17(3):335-43.

7. Ragheb S, Li Y, Simon K, VanHaerents S, Galimberti D, De Riz M, et al. Multiple sclerosis: BAFF and CXCL13 in cerebrospinal fluid. Mult Scler. 2011;17(7):819-29.

8. Hytonen J, Kortela E, Waris M, Puustinen J, Salo J, Oksi J. CXCL13 and neopterin concentrations in cerebrospinal fluid of patients with Lyme neuroborreliosis and other diseases that cause neuroinflammation. J Neuroinflammation. 2014;11:103.

9. Kowarik MC, Cepok S, Sellner J, Grummel V, Weber MS, Korn T, et al. CXCL13 is the major determinant for B cell recruitment to the CSF during neuroinflammation. J Neuroinflammation. 2012;9:93.

10. Brettschneider J, Czerwoniak A, Senel M, Fang L, Kassubek J, Pinkhardt E, et al. The chemokine CXCL13 is a prognostic marker in clinically isolated syndrome (CIS). PLoS One. 2010;5(8):e11986.

11. Krumbholz M, Theil D, Cepok S, Hemmer B, Kivisakk P, Ransohoff RM, et al. Chemokines in multiple sclerosis: CXCL12 and CXCL13 up-regulation is differentially linked to CNS immune cell recruitment. Brain. 2006;129(Pt 1):200-11.

12. Sellebjerg F, Bornsen L, Khademi M, Krakauer M, Olsson T, Frederiksen JL, et al. Increased cerebrospinal fluid concentrations of the chemokine CXCL13 in active MS. Neurology. 2009;73(23):2003-10.

13. Wang SM, Lei HY, Yu CK, Wang JR, Su IJ, Liu CC. Acute chemokine response in the blood and cerebrospinal fluid of children with enterovirus 71-associated brainstem encephalitis. J Infect Dis. 2008;198(7):1002-6.

14. Lepej SZ, Misic-Majerus L, Jeren T, Rode OD, Remenar A, Sporec V, et al. Chemokines CXCL10 and CXCL11 in the cerebrospinal fluid of patients with tick-borne encephalitis. Acta Neurol Scand. 2007;115(2):109-14.

15. Pranzatelli MR, Tate ED, McGee NR, Travelstead AL, Verhulst SJ, Ransohoff RM. Expression of CXCR3 and its ligands CXCL9, -10 and -11 in paediatric opsoclonus-myoclonus syndrome. Clin Exp Immunol. 2013;172(3):427-36.

16. Fragoso-Loyo H, Richaud-Patin Y, Orozco-Narvaez A, Davila-Maldonado L, Atisha-Fregoso Y, Llorente L, et al. Interleukin-6 and chemokines in the neuropsychiatric manifestations of systemic lupus erythematosus. Arthritis Rheum. 2007;56(4):1242-50.

17. Santer DM, Yoshio T, Minota S, Moller T, Elkon KB. Potent induction of IFN-alpha and chemokines by autoantibodies in the cerebrospinal fluid of patients with neuropsychiatric lupus. J Immunol. 2009;182(2):1192-201.

18. Franciotta D, Zardini E, Ravaglia S, Piccolo G, Andreoni L, Bergamaschi R, et al. Cytokines and chemokines in cerebrospinal fluid and serum of adult patients with acute disseminated encephalomyelitis. J Neurol Sci. 2006;247(2):202-7.

19. Matsushita T, Tateishi T, Isobe N, Yonekawa T, Yamasaki R, Matsuse D, et al. Characteristic cerebrospinal fluid cytokine/chemokine profiles in neuromyelitis optica, relapsing remitting or primary progressive multiple sclerosis. PLoS One. 2013;8(4):e61835.

20. Uzawa A, Mori M, Arai K, Sato Y, Hayakawa S, Masuda S, et al. Cytokine and chemokine profiles in neuromyelitis optica: significance of interleukin-6. Mult Scler. 2010;16(12):1443-52.

21. Fragoso-Loyo H, Cabiedes J, Richaud-Patin Y, Orozco-Narvaez A, Diamond B, Llorente L, et al. Inflammatory profile in the cerebrospinal fluid of patients with central neuropsychiatric lupus, with and without associated factors. Rheumatology (Oxford). 2009;48(12):1615-6.

22. Wang SM, Lei HY, Liu CC. Cytokine immunopathogenesis of enterovirus 71 brain stem encephalitis. Clin Dev Immunol. 2012;2012:876241.

23. Kamei S, Taira N, Ishihara M, Sekizawa T, Morita A, Miki K, et al. Prognostic value of cerebrospinal fluid cytokine changes in herpes simplex virus encephalitis. Cytokine. 2009;46(2):187-93.

24. Pranzatelli MR, Tate ED, McGee NR, Colliver JA. Cytokines, cytokine antagonists, and soluble adhesion molecules in pediatric OMS and other neuroinflammatory disorders. J Neurol Sci. 2013;326(1-2):53-8.

25. Neurath MF, Finotto S. IL-6 signaling in autoimmunity, chronic inflammation and inflammation-associated cancer. Cytokine Growth Factor Rev. 2011;22(2):83-9.

26. Dale RC, Morovat A. Interleukin-6 and oligoclonal IgG synthesis in children with acute disseminated encephalomyelitis. Neuropediatrics. 2003;34(3):141-5.

27. Ishizu T, Minohara M, Ichiyama T, Kira R, Tanaka M, Osoegawa M, et al. CSF cytokine and chemokine profiles in acute disseminated encephalomyelitis. J Neuroimmunol. 2006;175(1-2):52-8.

28. Uzawa A, Mori M, Sato Y, Masuda S, Kuwabara S. CSF interleukin-6 level predicts recovery from neuromyelitis optica relapse. J Neurol Neurosurg Psychiatry. 2012;83(3):339-40.

29. Uzawa A, Mori M, Ito M, Uchida T, Hayakawa S, Masuda S, et al. Markedly increased CSF interleukin-6 levels in neuromyelitis optica, but not in multiple sclerosis. J Neurol. 2009;256(12):2082-4.

30. Winter PM, Dung NM, Loan HT, Kneen R, Wills B, Thu LT, et al. Proinflammatory cytokines and chemokines in humans with Japanese encephalitis. Journal of Infectious Diseases. 2004;190(9):1618-26.

31. Goutieres F, Aicardi J, Barth PG, Lebon P. Aicardi-Goutieres syndrome: an update and results of interferon-alpha studies. Ann Neurol. 1998;44(6):900-7.

32. Fragoso-Loyo H, Atisha-Fregoso Y, Nunez-Alvarez CA, Llorente L, Sanchez-Guerrero J. Utility of interferon-alpha as a biomarker in central neuropsychiatric involvement in systemic lupus erythematosus. J Rheumatol. 2012;39(3):504-9.

33. Jonsen A, Bengtsson AA, Nived O, Ryberg B, Truedsson L, Ronnblom L, et al. The heterogeneity of neuropsychiatric systemic lupus erythematosus is reflected in lack of association with cerebrospinal fluid cytokine profiles. Lupus. 2003;12(11):846-50.

34. Wen SR, Liu GJ, Feng RN, Gong FC, Zhong H, Duan SR, et al. Increased levels of IL-23 and osteopontin in serum and cerebrospinal fluid of multiple sclerosis patients. J Neuroimmunol. 2012;244(1-2):94-6.

35. Maimone D, Gregory S, Arnason BG, Reder AT. Cytokine levels in the cerebrospinal fluid and serum of patients with multiple sclerosis. J Neuroimmunol. 1991;32(1):67-74.

36. Aurelius E, Andersson B, Forsgren M, Skoldenberg B, Strannegard O. CYTOKINES AND OTHER MARKERS OF INTRATHECAL IMMUNE-RESPONSE IN PATIENTS WITH HERPES-SIMPLEX ENCEPHALITIS. Journal of Infectious Diseases. 1994;170(3):678-81.

37. Ishizu T, Osoegawa M, Mei FJ, Kikuchi H, Tanaka M, Takakura Y, et al. Intrathecal activation of the IL-17/IL-8 axis in opticospinal multiple sclerosis. Brain. 2005;128(Pt 5):988-1002.

38. Obradovic D, Kataranovski M, Dincic E, Obradovic S, Colic M. Tumor necrosis factor-alfa and interleukin-4 in cerbrospinal fluid and plasma in different clinical forms of multiple sclerosis. Vojnosanit Pregl. 2012;69(2):151-6.

39. Drulovic J, Mostarica-Stojkovic M, Levic Z, Stojsavljevic N, Pravica V, Mesaros S. Interleukin-12 and tumor necrosis factor-alpha levels in cerebrospinal fluid of multiple sclerosis patients. J Neurol Sci. 1997;147(2):145-50.

40. Hauser SL, Doolittle TH, Lincoln R, Brown RH, Dinarello CA. Cytokine accumulations in CSF of multiple sclerosis patients: frequent detection of interleukin-1 and tumor necrosis factor but not interleukin-6. Neurology. 1990;40(11):1735-9.

41. Ichiyama T, Shoji H, Kato M, Sawaishi Y, Ozawa H, Matsubara T, et al. Cerebrospinal fluid levels of cytokines and soluble tumour necrosis factor receptor in acute disseminated encephalomyelitis. European Journal of Pediatrics. 2002;161(3):133-7.

42. Church AJ, Dale RC, Cardoso F, Candler PM, Chapman MD, Allen ML, et al. CSF and serum immune parameters in Sydenham's chorea: evidence of an autoimmune syndrome? J Neuroimmunol. 2003;136(1-2):149-53.

43. Dellalibera-Joviliano R, Dos Reis ML, Cunha Fde Q, Donadi EA. Kinins and cytokines in plasma and cerebrospinal fluid of patients with neuropsychiatric lupus. J Rheumatol. 2003;30(3):485-92.

44. Michael BD, Griffiths MJ, Granerod J, Brown D, Keir G, Wnek G, et al. The Interleukin-1 Balance During Encephalitis Is Associated With Clinical Severity, Blood-Brain Barrier Permeability, Neuroimaging Changes, and Disease Outcome. J Infect Dis. 2015.
